# Rapid Microchip Electrophoretic Separation of Novel Transcriptomic Body Fluid Markers for Forensic Fluid Profiling

**DOI:** 10.3390/mi13101657

**Published:** 2022-10-01

**Authors:** Tiffany R. Layne, Renna L. Nouwairi, Rachel Fleming, Haley Blair, James P. Landers

**Affiliations:** 1 Department of Chemistry, University of Virginia, Charlottesville, VA 22939, USA; 2 Institute of Environmental Science and Research Limited, Auckland 1025, New Zealand

**Keywords:** microfluidics, electrophoresis, messenger RNA, body fluid identification, forensic, fluorescence

## Abstract

Initial screening of criminal evidence often involves serological testing of stains of unknown composition and/or origin discovered at a crime scene to determine the tissue of origin. This testing is presumptive but critical for contextualizing the scene. Here, we describe a microfluidic approach for body fluid profiling via fluorescent electrophoretic separation of a published mRNA panel that provides unparalleled specificity and sensitivity. This centrifugal microfluidic approach expedites and automates the electrophoresis process by allowing for simple, rotationally driven flow and polymer loading through a 5 cm separation channel; with each disc containing three identical domains, multi-sample analysis is possible with a single disc and multi-sample detection per disc. The centrifugal platform enables a series of sequential unit operations (metering, mixing, aliquoting, heating, storage) to execute automated electrophoretic separation. Results show on-disc fluorescent detection and sizing of amplicons to perform comparably with a commercial ‘gold standard’ benchtop instrument and permitted sensitive, empirical discrimination between five distinct body fluids in less than 10 min. Notably, our microfluidic platform represents a faster, simpler method for separation of a transcriptomic panel to be used for forensically relevant body fluid identification.

## 1. Introduction

The identification of body fluids from forensic evidence provides context in a criminal investigation, informs downstream genetic analysis, and can corroborate witness testimony in court. However, body fluid identification (bfID) is often not straightforward, as many of the body fluids of interest are difficult to visually locate in a crime scene and are frequently present in small quantities or within mixtures. The majority of conventional bfID techniques rely upon time-consuming and laborious microscopic analysis or presumptive (bio)chemical, enzymatic, or immunological assays that are generally limited in specificity and lacking in sensitivity [1].

The conventional presumptive tests for bfID, e.g., phenolphthalein tetramethylbenzidine for blood, are unable to differentiate between closely related samples, such as menstrual and circulatory blood; the misidentification of these two fluids can significantly shift the narrative of a crime. As a result of this shortcoming, protein-based methods for bfID have emerged, such as machine learning methods based on the relative abundance of both marker and non-marker proteins [2], and spectroscopic-based tests (e.g., Raman [3] or Fourier transform infra-red spectroscopy [4]). However, these techniques are either remain presumptive in nature or limited by the requirement for expensive instrumentation, highly trained personnel, and the need for a library of peptide/protein sequences [5].

Recent advances in molecular biological approaches for confirmatory bfID include microbial community profiling, epigenetics, and transcriptomics. Microbial community profiling is based on the premise that body fluids exhibit different microfauna, but has primarily focused on the identification of vaginal secretions and saliva only [6]. Alternatively, epigenetic analysis identifies body fluids based on the varying methylation patterns that different cell or tissue types possess; however, shortcomings include requirements for high input DNA and susceptibility to change in response to environmental factors (aging, disease) [7,8]. As such, transcriptomics is arguably the most promising approach to bfID, with several different types of RNA under investigation, including messenger RNA [9,10], microRNA [11,12], and recently, circular RNAs [13,14]. A critical advantage of RNA profiling is that recovery of RNA from biological stains can be integrated into a typical forensic workflow without compromising or consuming the DNA that is essential for downstream genetic profiling and human identification.

The majority of methods for RNA-based bfID leverage reverse transcription polymerase chain reaction (RT-PCR), a sensitive method capable of detecting low-abundance mRNA obtained from small volume samples [9,15]. Although the most widely implemented approach utilizes end-point RT-PCR coupled with capillary electrophoresis (CE) [16,17], quantitative RT-PCR (RT-qPCR) can be employed for either relative quantitation from a reference or house-keeping gene, or for direct detection of a single amplicon using a DNA chelating dye or fluorescent probes [18,19]. Limitations of this RT-qPCR (e.g., non-specific annealing) have been ameliorated by high-resolution melting analysis [20] and involve a restricted number of markers that can be targeted in a single reaction. More recently, a method coupling reverse transcription with reverse transcription loop-mediated isothermal amplification (RT-LAMP) has been demonstrated, offering comparable sensitivity and specificity but is still limited to a single target per reaction [21,22]. Additionally, massively parallel sequencing for high-throughput bfID has been used in a number of academic studies, but the technique is expensive, time-consuming and requires extensive training, bioinformatics knowledge, and vast computer storage capabilities, ultimately limiting the real-world application [23,24,25]. Although a number of methods for bfID have been proposed, none are independently capable of providing a sample-to-result solution for reliable, accurate, and fast confirmatory bfID.

Microfluidic technologies are attractive for both lab-based and fieldable applications due to the inherently small footprint, minimal reagent/sample volume requirements, rapid analysis times, ease of use and, if needed, portability [26,27]. Significant advances have been made through research and development of microfluidic devices for a range of applications, including clinical (e.g., virus detection [28], biomolecule cleaning [29], etc.) and forensic (STR profiling [30], epithelial and sperm separation, etc.) applications. However, limitations in adopting microfluidic systems often stem from failing to address the costly nature of the microfluidic consumable [31]. Our lab has defined a cost-effective method for rapid, iterative prototyping of microfluidic CDs (print-cut-laminate technique; PCL [32]) with complex, intricate architectures for chemical and biochemical assays ranging from DNA purification and genome analysis to illicit drug detection and explosives sensing [33,34,35].

By leveraging the PCL technique, we are able to rapidly prototype cost-efficient microdevices for electrophoretic separation and fluorescent detection of mRNA amplicons associated with forensically relevant body fluids. The resultant system includes a centrifugal microelectrophoresis Disc (*μ*EDisc), custom separation polymer, and a mechatronic system [36] to apply voltages for electrokinetic, size-based separation and perform laser-induced fluorescence (LIF) detection of PCR amplified fragments. Using this platform, the microchip electrophoretic analysis time is decreased 4-fold over conventional CE. Here, we demonstrate successful electrophoretic separation of mRNA targets from saliva, venous blood, menstrual blood, vaginal fluid, semen, and seminal fluid as individual and mixed samples. Resultant profiles from our *μ*EDisc exhibit concordance with profiles generated from conventional, benchtop capillary electrophoresis (CE).

## 2. Materials and Methods

### 2.1. Sample Collection

Body fluid samples were collected from volunteers with fully informed consent. Blood was collected via fingerprick, where 50 µL was placed on sterile Cultiplast^®^ rayon swabs (LP Italiana SPA, Milano, Italy) and left to dry overnight. Buccal, vaginal fluid, and menstrual fluid (day 2 and 3 of the menstrual cycle) samples were collected from the volunteers themselves using sterile Cultiplast^®^ rayon swabs and the swabs left to dry overnight. Semen samples were collected from the volunteers themselves in sterile plastic containers, then 50 µL aliquots were placed on sterile Cultiplast^®^ rayon swabs and left to dry overnight.

### 2.2. RNA Isolation

The RNA was isolated as previously described [37]. Total RNA for all samples was extracted using the Promega^®^ ReliaPrep™ RNA Cell Miniprep System (Promega Corporation, Madison, WI, USA) following the manufacturer’s instructions. DNA was removed from extracted RNA using an on-column DNase I treatment during the RNA extraction process. RNA was eluted in 70 µL of elution buffer. Purified RNA samples were further treated with DNase immediately after extraction using the TURBO DNA*free*™ Kit (Ambion, Inc. Austin, TX, USA). The manufacturer’s instructions were followed, adding 7.0 μL 10× TURBO DNase Buffer and 2 μL TURBO™ DNase to each sample. Complete removal of human DNA was verified using the Quantifiler^®^ Human DNA quantification kit (Life Technologies Corp., Carlsbad, CA, USA) using 1 μL of sample in a 12.5 μL reaction. The RNA was stored at −80 °C until required.

### 2.3. Primer Information

The primer sequences for eleven mRNA targets were published by Albani et al. [38]. These targets are divided into three multiplexes specifically identifying six body fluids (Table S1). A single primer for each target is tagged with either FAM or HEX and all targets create a fragment that is less than 200 bp in length.

### 2.4. RT-PCR Method

RNA samples (5 μL) were reverse transcribed using the High-Capacity cDNA Reverse Transcription Kit (Applied Biosystems, Waltham, MA, USA) according to the manufacturer’s instructions. Each reaction comprised a total volume of 20 µL. For mixed body fluid samples, vaginal and buccal cDNA samples were diluted by 1/10 using nuclease-free water. The semen cDNA was diluted by half using nuclease-free water. For the vaginal/buccal mixed sample, diluted vaginal cDNA (2 µL) was added to 1 µL of the buccal diluted cDNA. The vaginal/semen mixed sample contained diluted vaginal cDNA (2 µL) and 1 µL of the semen diluted cDNA.

PCR was performed on a GeneAmp PCR System 9700 (ThermoFisher, Waltham, MA, USA) in 25 μL reactions using 12.5 μL Qiagen^®^ Multiplex PCR buffer (Hilden, Germany), 2.5 μL primer mix, and 3 μL cDNA. The total reaction volume of 25 μL was achieved by the addition of 7 μL nuclease-free water. The PCR conditions were initial denaturation at 95 °C for 15 min, followed by 35 cycles of 94 °C for 30 s, 60 °C for 3 min and 72 °C for 1 min, final elongation at 72 °C for 10 min, and cooling down to 4 °C.

### 2.5. Capillary Electrophoresis

The amplified samples were heated to 95 °C for 3 min and snap-cooled on ice for 3 min prior to electrophoresis. Separation was conducted on a 3130XL capillary electrophoresis instruments (Applied Biosystems, Waltham, MA, USA) using 1 µL amplified sample and 10 µL Internal Lane Standard (1500 kb) and Hi-Di formamide. The samples were injected for 5 s at 3 kV and analyzed using GeneMarker HID v.2.8.2 (SoftGenetics, LLC, State College, PA, USA).

### 2.6. Microchip and Fabrication

The centrifugal microfluidic disc for DNA separation and detection was developed using the print–cut–laminate (PCL) method described by Thompson et al. [32]. Fabrication and assembly of the electrophoresis microfluidic architecture was triplicated on a single *μ*EDisc, which measured 13.2 cm in diameter. Each *μ*EDisc was assembled using five polymeric layers, including polyethylene terephthalate (PeT; TRANS-NS, Filmsource Inc., Maryland Heights, MO, USA), black PeT (bPeT, TRANS-NS, Filmsource Inc., Maryland Heights, MO, USA), and a heat-sensitive adhesive (HSA; Adhesives Research, Inc., Glen Rock, PA, USA). All layers were laser ablated using a CO_2_ laser system (VLS3.50, Universal^®^ Laser Systems, Scottsdale, AZ, USA) to transmit microfluidic architecture. The layers were washed using two 10 min cycles of deionized water and dried in a chemical hood overnight. The layers were aligned and heat-bonded by passing the aligned disc through an office laminator heated to 180 °C. Lastly, each domain contained an injection molded cyclic olefin copolymer (COC) microchip bonded to the underside of the *μ*EDisc through a pressure sensitive adhesive (PSA; Adhesives Research, Inc., Glen Rock, PA, USA) in which electrophoretic separation of complementary DNA (cDNA) fragments transpired; the separation microchip contained an electrophoresis channel with 50 µm × 50 µm channels (W × H) with a 5 cm effective separation length (L_eff_) To complete the electrophoretic circuit for separation, gold electrodes were fabricated from 24K gold sheets (L.A. Gold Leaf Wholesalers, Covina, CA, USA) bonded to PSA and PeT, then laser-ablated to achieve the correct architecture prior to bonding on top of the *μ*EDisc. The *μ*EDisc measured 133 mm in diameter and 0.6–3.5 mm in height depending on the features. The 3D-printed adapter piece for interfacing the *μ*EDisc with the centrifugal platform was made of black EverydayPLA (polylactic acid; Type A Machines, CA, USA) filament using a Series 1 PRO (Type A Machines, San Leandro, CA, USA). The completed adapter measured 46 mm in diameter with an inner hole of 13 mm diameter and 1.4 mm in height.

### 2.7. Micro-Electrophoresis and Data Analysis

The automated mechatronic system, described elsewhere, was connected to a PC laptop containing custom software to control all the necessary movements of the *μ*EDisc as well as data collection and adaptation for analysis in GeneMarker HID software [36,39]. Sample input volumes, heating during separation, and injection times were varied to optimize electrophoresis conditions. The appropriate sample volume was determined by adding either 1, 2, or 3 µL of sample to 3 µL ILS and nuclease-free water to bring the total reaction volume to 13 µL. For heating studies, a metal stage in contact with the underside of the COC chip was either heated to 37 °C throughout separation via a Peltier heater or the stage was not heated during separation, and injection times were varied using 60, 90, 120, and 180 s. Prior to separation, a novel hydrophobically modified polyacrylamide polymer [40] was centrifugally loaded via device rotation at 3000 RPM for 5 min, followed by the actuation of two laser valves [41] (described in Woolf et al.) to establish electrical connectivity with the polymer in the sample waste (SW) and buffer (B) electrodes. A third laser valve was actuated and the *μ*EDisc was spun at 3000 RPM for 10 s to allow the sample to interface with the sample (S) electrode. Off disc, the sample was heated at 95 °C for 3 min then snap cooled on ice for 3 min then 3 µL of the cDNA was added to 3 µL WEN ILS 500 (Promega, Madison, WI, USA) and 6.5 µL nuclease-free water (Molecular Biologicals International, Inc., Irvine, CA, USA) prior to loading into the *μ*EDisc. Once fluidic movement was complete, the *μ*EDisc separation channel was located rotationally via the photo-interrupter and clamped by the clamping motor to establish contact between the gold pogo pins with the gold electrode pads on the *μ*EDisc. Electrokinetic injection was performed at 600 V for 120 s from S to SW electrodes, and separation at 1500 V from B to BW with a 200 V pullback voltage applied at S and SW for 500 s. The effective length of separation (cross-t to detector) is 6 cm. At completion of the separation, integrated data analysis was performed in the software through a data analysis pipeline, which involves trimming of primer peaks, baseline subtraction, pullup correction, and 10× signal amplification using a digital filter. The processed data were then re-formatted and saved as a “.txt” file for compatibility with Microsoft Excel, as well as a “.fsa” file for forensic analysis in GeneMarker HID software (V2.8.2; SoftGenetics, LLC., State College, PA, USA) [42]. The *μ*EDisc and automated system were used to separate all of the single- and multiple-body fluid samples amplified with one of the three body fluid panels.

## 3. Results and Discussion

In previous work, Albani et al. optimized three bfID panels consisting of eleven mRNA genes for identifying five forensically relevant body fluids (Table S1) [38]. The Duplex Panel consists of FDCSP (170 bp; HEX) and HTN3 (138 bp; HEX) to identify buccal mucosa or saliva. The Quadruplex Panel consists of HBD (176 bp; FAM) and SLC4A1 (102 bp; HEX) to identify venous blood, and MMP10 (108 bp; HEX) and STC1 (105 bp; FAM) to identify menstrual blood. The Pentaplex Panel consists of PRM1 (150 bp; HEX) and TNP1 (102 bp; FAM) to identify spermatozoa, KLK2 (135 bp; HEX) and MSMB (142 bp; FAM) to identify seminal fluid, and CYP2B7P (113 bp; HEX) to identify vaginal fluid. For each panel, resultant amplicons can be electrophoretically separated and detected using fluorescently labeled primers (FAM and HEX). All PCR amplicons were less than 200 base pairs (bp) in length, and widely published in the forensic literature whereby analysis was typically performed using conventional capillary electrophoresis (CE) instrumentation [17,43]. Conversely, we describe the adaption of such assays onto a microfluidic electrophoresis disc (*μ*EDisc) capable of electrokinetic separation and LIF detection of these forensically relevant mRNA targets.

### 3.1. Microfluidic Disc and Integrated System

**Figure 1:**
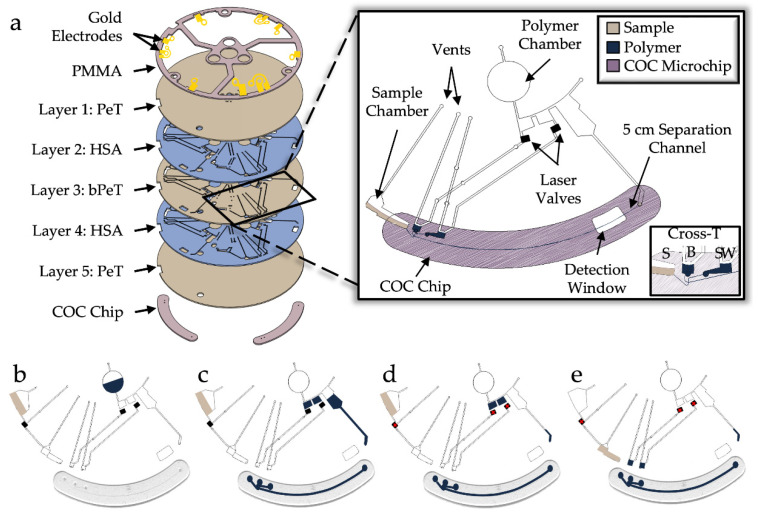
Design and architecture of *μ*EDisc for electrokinetic separation of amplified forensically relevant fragments. (**a**) An exploded view of the 5 layer microfluidic disc, comprising polyethylene terephthalate (PeT), heat sensitive adhesive (HSA), and black PeT with accessory pieces on the top and bottom of the disc, including poly(methyl methacrylate) (PMMA), gold electrodes, and an injection molded cyclic olefin copolymer (COC) separation chip. The inset shows a labeled schematic of an individual domain in the disc with a close-up of the separation cross-T and the sample (S), buffer (B), and sample waste (SW) reservoirs. Fluid was rotationally driven through the architecture in four simple steps: (**b**) sample and polymer were pipetted into the respective chambers and (**c**) polymer was centrifugally metered. (**d**) Laser valves were then opened (as indicated by a red ‘X’), and (**e**) the disc was spun to rotationally drive fluid into the S, B, and SW reservoirs.

The microfluidic disc was fabricated using the cost-effective Print, Cut, and Laminate method, previously described [32]. The *μ*EDisc consisted of three identical domains for electrophoresis, each containing chambers for separation polymer and sample, enclosed reservoirs for sample (S), buffer (B), sample waste (SW), and buffer waste (BW), as well as an injection molded COC chip containing the separation cross-T and channel (Figure 1a). The electrophoresis microchip has an effective separation length of 5 cm with ~1 cm arms connecting the cross-T to the the S, B, and SW reservoirs. Each domain has gold electrodes capping the S, B, SW, and BW reservoirs to interface a voltage power supply with hermetically sealed fluid. To perform electrophoresis with this microdevice, sample/reagents and separation polymer are pipetted into the Sample Chamber and Polymer Chamber, respectively (Figure 1b). Polymer is then rotationally driven into two metering chambers and through the COC chip containing the separation cross-T (Figure 1c). Valves are opened via laser actuation to allow the metered polymer to be centrifugally driven to the B and SW reservoirs (Figure 1d). Finally, the last laser valve is opened to enable the sample/reagents to flow to the S reservoir before electrophoresis can begin (Figure 1e).

**Figure 2:**
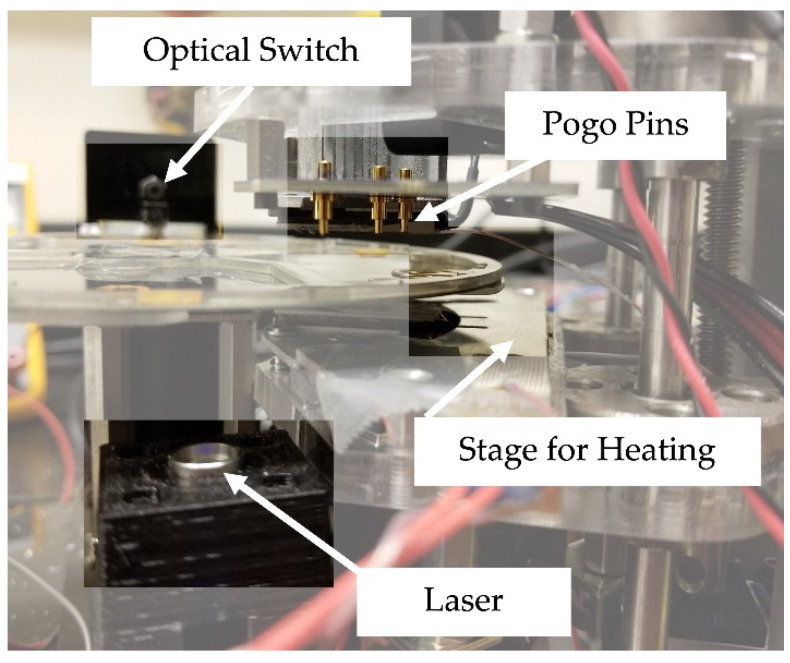
The automated system for microchip electrophoresis has a laser for opening valves, a stage that sits along the separation channel for heating, an optical switch for alignment, and pogo pins to apply voltages to electrodes on the disc during electrophoresis.

An instrument capable of electrophoresis in a polyethylene disc with the corresponding software was developed to allow for automated microfluidic steps on a centrifugal microfluidic disc [36]. The same instrument and software were repurposed to permit flow and produce results with the mRNA targets in the body fluid panels on the *μ*EDisc. Briefly, the disc is mounted onto a rotational platform, much like a CD-ROM, and an optical sensor placed near the outer perimeter of the disc allows for accurate positioning of the *μ*EDisc (Figure 2). The motor driving the rotational platform is controlled by software (Atmel Studio v7.0; Microchip Technology Inc. Changler, AZ, USA) where the spin speed is defined by user input. The system associates architectural features with coordinates that are defined by: (1) the distance from the center of the disc, and (2) the number of degrees from ‘home’ (which is 0°); these are input to the software to align the red laser diode with valves that are opened by ablation. Similarly, defined by coordinates relative to ‘home’, the system can rotate the disc to the accurate position needed to align the gold POGO pins for contact with the gold electrodes for application of voltage to the separation microchannel. Specific voltage/times are used to define consistent injection at the cross-t followed by size-based separation of the fragments. The detection system is equipped with a blue laser (488 nm) for excitation, with emitted light funneled by the optics to a four-color optical detector (photomultiplier tube) as fragments pass through the detection window.

### 3.2. Optimizing Separation Parameters

Unlike commercial capillary electrophoresis (CE), the development of CE on novel systems such as the one described herein inherently requires that a number of parameters be optimized for effective functioning of the system [44]. Previous work has shown sufficient separation of 10 STR loci ranging from 80 to 500 bp using this on-disc platform [30,36], thus, in this study we focused on optimizing sample input, heating of the separation channel, and sample injection time to adequately separate the amplicons in three body fluid panels.

When compared to gold standard instrumentation, where the electrokinetically injected sample contains amplified fragments, internal lane standard (ILS), and hi-di formamide, the sample injected on the *μ*EDisc system contains amplified fragments, ILS, and water. While this difference (water versus formamide) may cause concern, previous work has shown this has no adverse effects on the size-dependent separation on-disc [42]. The ratio of sample volume to injection total volume is doubled on this system; in other words, 1 µL of amplified sample goes into 9 µL of Hi-Di formamide and ILS on a conventional CE system, whereas 3 µL amplified sample goes into 10 µL of water and ILS on this system. Due to differences between conventional CE and this system, the parameters for electrophoresis (i.e., sample volume, heating during separation, and injection time) should be optimized to show sufficient size separation of the amplified fragments.

To assure optimal conditions for separation of the fragments, we assessed the height, size, and shape of the fluorescent signal, or peaks, for three different fluorescent channels: the blue and green channels detected the alleles, and the red channel detected the ILS.

**Figure 3:**
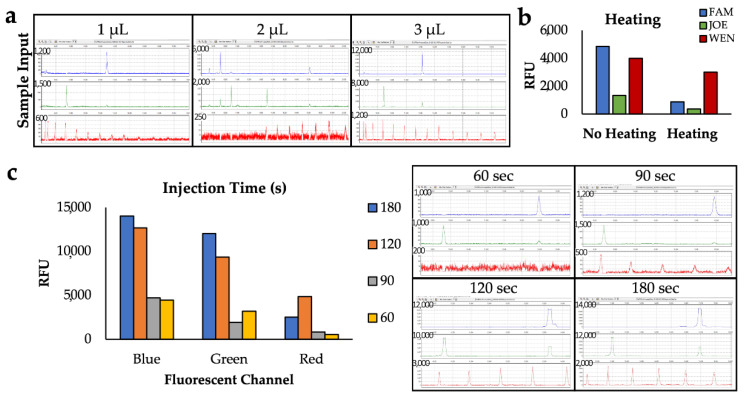
Optimization of separation parameters through comparison of electrophoresis peaks. (**a**) Resulting electropherograms from reagents containing 1, 2, or 3 µL of sample in a 13 µL sample volume. (**b**) Electrophoresis peak heights for each fluorescent channel in terms of relative fluorescence units (RFU) comparing results when the separation channel is heated to 37 °C or not heated. (**c**) Electropherograms showing the difference in peaks when injecting for 60, 90, 120, or 180 s, and a bar graph comparing the peak heights in terms of relative fluorescent units.

While 3 µL of amplicons added to 10 µL of water and 3 µL ILS for a total volume of 13 µL was previously demonstrated to be sufficient for separation of amplified STR fragments [42], here we analyzed a smaller input of amplified sample in the same total volume to determine if a similar input volume as the conventional CE system could be used on-disc (Figure 3a). However, a 3 µL volume was shown to yield high fluorescent signal for both the blue and green channels with peak heights decreasing as input sample volumes were reduced. Higher input volumes were considered, but we wanted to balance using the least volume of sample while obtaining peak heights that are high, but do not saturate the detector. Therefore, we selected to use a sample cocktail comprising 3 µL of sample, 4 µL of ILS, and 6 of µL water as this yields the most robust detection of both ILS peaks and sample amplicons peaks.

The optimal channel temperature was assessed by heating the stage under the separation chip to 37 °C throughout electrophoresis or omitting heating on the *μ*EDisc. Since the capillaries on conventional systems are heated by an oven during separation, it was hypothesized that heating of the COC channel on the *μ*EDisc would be advantageous. It was important to balance the peak height of the targets in the blue and green channels with high peaks in the red channel. Results showed that heating during a separation caused the peak heights to decrease (Figure 3b). It was theorized that there was a heat gradient vertically through the channel that could impede the separation due to a change in viscosity of the polymer. Alternatively, the heat produced from the voltage through the capillary combined with extra heating from the metal plate could cause joule heating affecting the resolution of the peaks, leading to smaller peak heights. Due to these possibilities and the experimental data, the metal plate was not heated during electrophoresis.

Finally, optimization of sample injection time was seminal in balancing maximum peak height with minimal peak broadening. The electrokinetic injection was performed by applying 600 V to the S and SW electrodes, since this voltage was previously shown to give successful separations on a similar system [39]. If the injection time is not long enough, the sample will not reach the cross-t for sufficient fluorescent detection; on the other hand, if the injection time is too long, the bulk of the sample may bypass the cross-t leading to decreased fluorescent signal. Figure 3c shows a representative resulting electropherogram from various injection times, including 60, 90, 120, and 180 s. With a 60-s injection, the amplicon peaks associated with mRNA targets and ILS peaks were not present. However, using a 90-s injection the peaks associated with amplicons of mRNA targets were present and ILS peaks were detected with lower peak heights over time. Detection of the ILS peaks, especially in the 80–200 base pair range, is critical for accurate sizing of the amplicon peaks since all of the mRNA targets in the panel range from 100 to 200 bp in size. Of all the injection times, the 120- and 180-s injections were the only two that detected the ILS peaks well after the 200-base pair peak and all of the sample peaks associated with mRNA targets; the corresponding electropherograms show peaks in the FAM and HEX channels saturating the detector (as indicated by the flat, cut-off peak). While either injection time could be used to detect all the necessary ILS peaks with the on-disc electrophoresis, we selected 120-s injection, ultimately decreasing overall separation time by one minute, making the overall separation time 10 min (i.e., a 2 min injection facilitated by 600 V applied to the S and SW reservoirs followed by an 8 min separation via application of 1500 V across the 5 cm electrophoresis channel).

### 3.3. Single Source Fluid Comparison

Once the on-disc electrophoresis was optimized for amplicon separation, separation of all body fluids was examined and the microfluidic data were compared to a commercial ABI 3130XL. Due to the differences in instrument sensitivity, the resultant peak heights, in terms of relative fluorescent units (RFU), were minimum-maximum normalized for each detection system to more accurately compare between the separation platforms (Figure S1) [45].

**Figure 4:**
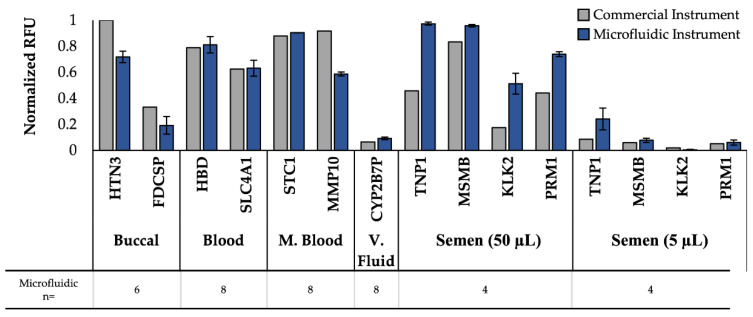
Single source fluid comparison of normalized peak heights from a commercial CE instrument and the microfluidic system.

Using this normalization, the normalized peak heights for each body fluid mRNA target were compared for commercial and microfluidic instruments (Figure 4). It is worth noting that samples separated on the commercial instrument were only analyzed replicates of 1 as this is often standard practice in forensic laboratories due to limited starting sample volumes, but multiple replicates for microfluidic data were run. The 11 peaks associated with mRNA targets produced following amplification via the three panels (Duplex, Quadruplex, and Pentaplex) were consistently detected in the samples separated on the *μ*EDisc, showing comparable results with respect to peak height with the commercial instrument; variability in RFUs for each allele are expected as samples were collected from different donors. These results are significant because not only did the *μ*EDisc detect all of the gene peaks associated with amplicons of the mRNA targets, but in some of the body fluids the *μ*EDisc detected the peaks as well or better than the commercial systems. For example, peaks associated with amplicons of both mRNA targets specific to venous blood (HBD and SLC4A1) were detected with the microfluidic system in all samples with comparable intensity relative to the commercial instrument. These samples consistently showed peak heights from the *μ*EDisc that were higher than or similar to the commercial instrument. Similar trends were observed when considering the two peaks associated with menstrual blood targets (STC1, MMP10). As expected, the two peaks associated with venous blood targets (HBD, SLC4A1) were also detected in both menstrual blood samples, but in much lower peak heights using the *μ*EDisc. This demonstrates that the *μ*EDisc can allow for detection of multiple amplicons with high and low fluorescent signal.

The duplex peaks associated with amplicons of mRNA targets specific to buccal (HTN3, FDCSP) were consistently detected in both systems in multiple amplified samples from buccal swabs. Figure 4 shows the HTN3 peak to be consistently higher than the FDCSP peak using the *μ*EDisc system, which was also found in the commercial system.

Finally, the Pentaplex panel containing the vaginal fluid and semen mRNA targets was detected on both separation platforms using two vaginal fluid donors and two semen donors (50 µL and 5 µL). While the vaginal fluid marker (CYP2B7P) was one of the lowest normalized peak heights detected across both platforms, the peak height was consistently detected using the *μ*EDisc system (Figure S2). Higher peak heights were observed for CYP2B7P on the *μ*EDisc system than the commercial system, showing the *μ*EDisc platform compares well with the gold standard instrument. Similarly, all four peaks associated with amplicons of semen mRNA targets (TNP1, MSMB, KLK2, PRM1) were detected using the *μ*EDisc system at higher or comparable peak heights than the commercial instrument with the 50 µL semen sample, again showing a strong comparison between the *μ*EDisc system and the gold standard instrument. This trend continued with the 5 µL semen sample, with the exception of the KLK2 gene, which was manually called with a peak height of 845 RFU using the *μ*EDisc system. These data show that even though the sample was small in volume, the *μ*EDisc system was still able to detect all of the semen genes in the Pentaplex panel in 10 min. Additionally, the data show that all of the vaginal fluid and semen samples were detected in the *μ*EDisc platform.

### 3.4. Multiple Source Fluid Comparison

**Figure 5:**
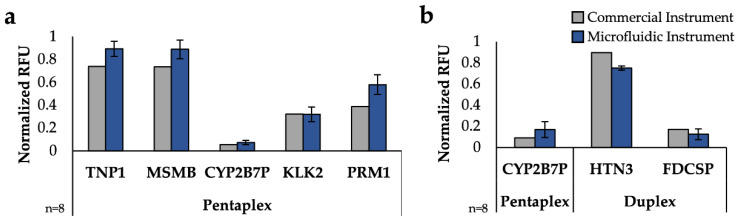
Multi-source fluid comparison of peak heights from gold-standard CE systems and the microfluidic system. (**a**) Electrophoresis of contrived mixed sample containing vaginal fluid and semen amplified with the Pentaplex panel of primers. (**b**) Normalized peak heights from a mixed sample containing vaginal fluid and saliva amplified with the Pentaplex and Duplex primers.

While detection of single-source fluids is important, many forensic samples are not single source; they can contain anywhere from one fluid to all five fluids, plus others not tested here. To further test the size separation of the panels on the *μ*EDisc, the amplified products from two mixed samples were separated on both platforms for comparison. To simulate types of samples that would be present at a crime scene, swabs containing vaginal fluid were spiked with semen or saliva and amplified using the Pentaplex or Duplex panel of primers (Figure 5). Figure 5a shows simultaneous detection of all 5 Pentaplex targets from a swab containing vaginal fluid and semen using the commercial instrument and the microfluidic system. The semen target (TNP1) and seminal fluid target (MSMB) amplicons were detected using the *μ*EDisc system at peak heights higher than the commercial system after normalization, while the other three genes in the panel for vaginal fluid (CYP2B7P), seminal fluid (KLK2) and semen (PRM1) had comparable peak heights between instruments. The second mixture sample comprised vaginal fluid and buccal fluid and was amplified with the Duplex and Pentaplex panels (Figure 5b). The buccal amplicon (HTN3) was detected on the *μ*EDisc with lower peak heights compared to the commercial instrument, while the vaginal fluid (CYP2B7P) and buccal (FDCSP) genes exhibited similar peak height between platforms. While there is variation in peak heights between the two platforms (which is expected as samples were collected from different donors at different times), the key takeaway is that there was successful separation, detection, and identification of all body fluids present in the contrived samples. These data show the *μ*EDisc system can perform just as well as or better than the gold standard systems with single- and multiple-body fluid samples. It also shows replicate consistency in the *μ*EDisc system among both types of samples.

### 3.5. On-Disc Detection Sensitivity

**Figure 6:**
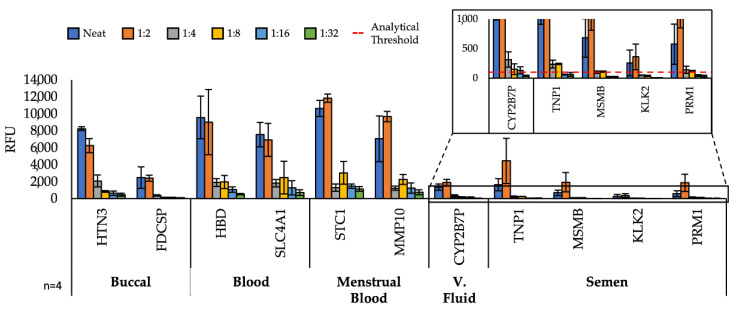
Separation of all single-source body fluids to determine the sensitivity of on-disc fluorescent detection. Amplified neat samples were serially diluted down to a 1:32 dilution to examine the sensitivity of the microfluidic platform. The inset shows a closeup of the Pentaplex panel of targets and the analytical threshold (100 RFU) of the *μ*Edisc.

We sought to interrogate the sensitivity of our *μ*EDisc system by empirically determining an analytical limit of detection for each body fluid panel. Any analytical system has thresholds that differentiates an actual peak from background noise. For the *μ*EDisc system, we determined the analytical threshold by taking 10% of the gene with the lowest peak height from a run. Each PCR product was serially diluted from the neat sample to a 1:32 factor and electrophoresed on the microfluidic system to simulate low-concentration samples frequently encountered in forensic casework (Figure 6). Unsurprisingly, after a 1:2 dilution, the fluorescence signal considerably decreased for all amplicons in the three panels. Even with the decrease in signal, 10 of the 11 genes were automatically detected by the GeneMarker software at the 1:32 dilution. The KLK2 gene was the only one that completely dropped below the analytical threshold of the microfluidic platform (100 RFU) after a 1:4 dilution of the 5 µL semen sample (Figure 6 inset). Further, the vaginal fluid gene (CYP2B7P) was detected at the lowest dilution of 1:32 using the *μ*EDisc system. Using both our guidelines (threshold and detection of all body fluid amplicons), the sensitivity of the *μ*EDisc system for semen is a 1:2 dilution of a 5 µL sample and for vaginal fluid, a 1:16 dilution of a whole swab sample.

Similarly, the sensitivity of targets associated with the quadruplex panel showed that after a 1:2 dilution there was a sharp decline in fluorescent signal, but even at the 1:32 dilution both genes for venous blood and menstrual blood were detected above the analytical threshold using the *μ*EDisc system. Using the same standards, the *μ*EDisc system for venous blood and menstrual blood is a 1:32 dilution of liquid or whole swab sample, respectively.

Finally, the two buccal targets in the Duplex panel (HTN3, FDCSP) were detected with the *μ*EDisc system. The peak associated with the amplicon of mRNA target HTN3 was detected well above the threshold, but peaks associated with the FDCSP amplicon were only detected above the threshold up to the 1:8 dilution. For the Duplex panel, the sensitivity of the *μ*EDisc system for buccal fluid is a 1:8 dilution of a whole swab sample.

## 4. Conclusions

This research showed proof-of-concept microfluidic disc electrophoresis as part of a transcriptome-based method for forensic bfID. Given that forensic laboratories are overburdened, it was important to achieve this technique in a rapid system. To address this, the *μ*EDisc was designed to accommodate three parallel domains to enable simultaneous analysis of three samples in approximately 10 min. Importantly, all mRNA markers for single-source samples of five body fluids, as well as mixed samples, were detected using the three primer panels.

Optimization herein demonstrated that a 120 s injection of a 3 µL sample volume without heating the channel were the best conditions for separating the amplicons produced from genetic targets specific to various (5) body fluids. Using these conditions, all 11 genes from the three body fluid panels were detected consistently on the *μ*EDisc. This is significant because it shows the microfluidic platform can detect all 11 genes in a fraction of the time consumed on commercial systems. Lastly, the sensitivity of the *μ*EDisc system was tested with dilutions of the single-body fluid samples. While each of the body fluids had a different sensitivity on the *μ*EDisc system, the mRNA genes were consistently auto called in all of the dilutions, with the exception of the KLK2 gene.

Finally, these results demonstrate the feasibility for future development of a fully integrated *sample in–answer out* system through incorporation of on-disc RNA extraction and RT-PCR amplification. Such a device would have important implications in expediting the forensic body fluid identification process with high sensitivity and specificity of mRNA targets. To realize this, the next steps will be to adapt the RT-PCR amplification previously used with these panels onto the microdevice. The existing *μ*EDisc architecture will be augmented to accommodate RNA extraction and RT-PCR without altering device fabrication methods. The resulting miniaturized total analysis system (µTAS) will permit complete, automated, direct-from-sample RNA-based body fluid identification amenable for use by nontechnical personnel. Overall, this research shows progress towards a new body fluid detection method that can rapidly and consistently identify any of the five forensically relevant body fluids.

## Data Availability

The data presented in this study are available on request from the corresponding author.

## Supplementary Materials

The following supporting information can be downloaded at: https://www.mdpi.com/article/10.3390/mi13101657/s1, Table S1. Three multiplexed panels of forensically relevant body fluid targets were designed and validated by Albani et al. [36]. Each panel comprises fluorescently tagged messenger RNA primers that are specific for each fluid and create fragments that are less than 200 bp, Figure S1. Process of minimum–maximum normalization to compare peak heights in terms of relative fluorescence units (RFU) across different electrophoresis platforms, Figure S2. Representative electropherograms from single-source body fluid samples after microchip electrophoresis. Electropherograms are grouped based on the three panels of mRNA targets.
