# Supplementary material for: Rapid Microchip Electrophoretic Separation of Novel Transcriptomic Body Fluid Markers for Forensic Fluid Profiling
Source: Micromachines (Basel). 2022 Oct 1;13(10):1657. doi: 10.3390/mi13101657 (PMC9609788; doi:10.3390/mi13101657)
Supplement: Supplementary file 1 [file micromachines-13-01657-s001.zip › micromachines-1903735-supplementary.pdf]

Table S1. Three multiplexed panels of forensically-relevant body fluid targets were designed and validated by Albani *et al.* [36]. Each panel is comprised of fluorescently-tagged messenger RNA primers that are specific for each fluid and create fragments that are less than 200 bp.

Multiplex	Target Body Fluid	mRNA	Primer Sequence	Final Concentration in PCR assay (μM)
Duplex	Saliva / Buccal	FDSCP	F- HEX-CTCTCAAGACCAGGAACGAGAA<br>R - GGGCAGATTCAGGTATTGGAATAG	0.05
		HTN3	F - HEX/AAGCATCATTCACATCGAGGCTAT<br>R - ATGCGGTATGACAAATGAGAATACAC	0.03
Quadruplex	Circulatory Blood	HBD	F - ACTGCTGTCAATGCCCTGTG<br>R - FAM/ACCTTCTTGCCATGAGCCTT	0.05
		SLC4A1	F - HEX/AACTGGACACTCAGGACCAC<br>R - GGATGTCTGGGTCTTCATATTCCT	0.04
	Menstrual Fluid	MMP10	F - HEX/CCCACTCTACAACTCATTCACAGAG<br>R - GGTTCCTCAGTAGAGGCAGG	0.04
		STC1	F - FAM/CTGCCCAATCACTTCTCCAACA<br>R - TTTCTCCATCAGGCTGTCTCT	0.02
Pentaplex	Spermatozoa	PRM1	F - HEX/GCCAGGTACAGATGCTGTCGCAG<br>R - GTGTCTTCTACATCTCGGTCTG	0.03
		TNP1	F - GATGACGCCAATCGCAATTACC<br>R - FAM/CCTTCTGCTGTTCTTGTTGCTG	0.04
	Seminal Fluid	KLK2	F - TTCTCTCCATCGCCTTGTCTG<br>R - HEX/AGTGTGCCCATCCATGACTG	0.14
		MSMB	F - CTTTGCCACCTTCGTGACTTTATG<br>R - FAM/ACAGTTGTCAGTCTGCCACT	0.03
	Vaginal Material	CYP2B7P	F - CCGTGAGATTCAGAGATTTGCTGAC<br>R - HEX/TGAGAAATACTTCCGTGTCCTTGG	0.02

$$Normalized\ RFU = \frac{RFU\ of\ gene - Lowest\ RFU}{Highest\ RFU\ - Lowest\ RFU}$$

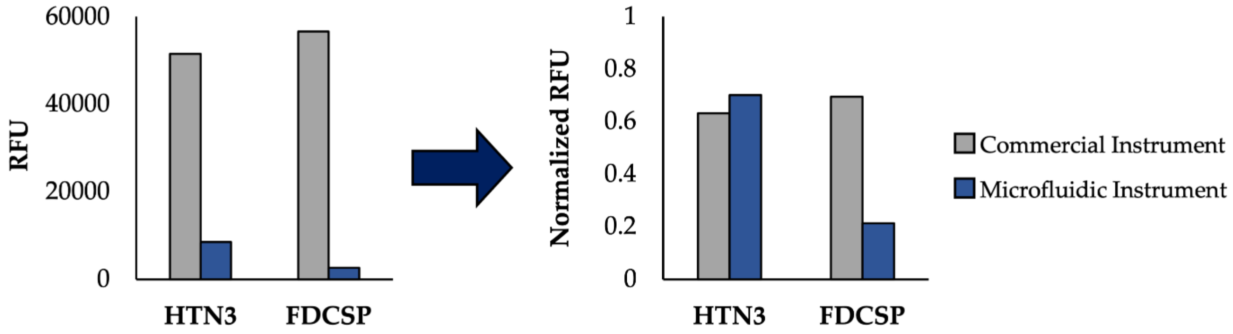

Figure S1. Process of minimum-maximum normalization to compare peak heights in terms of relative fluorescence units (RFU) across different electrophoresis platforms.

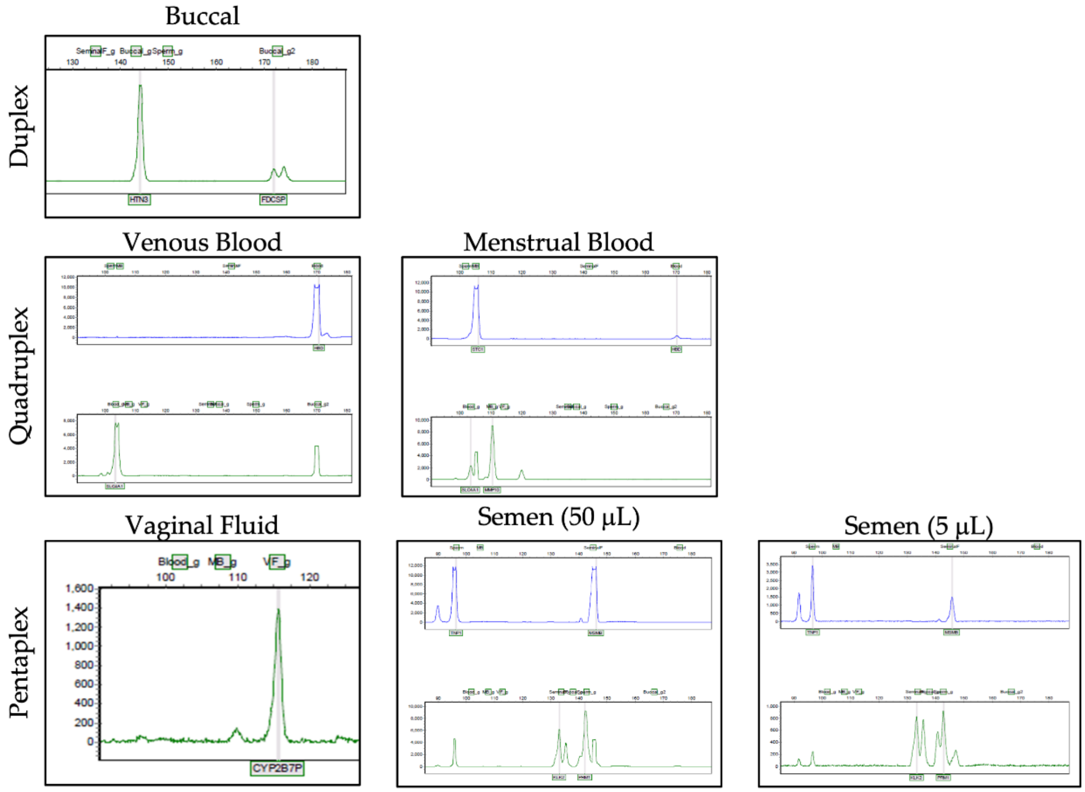

Figure S2: Representative electropherograms from single-source body fluid samples after microchip electrophoresis. Electropherograms are grouped based on the three panels of mRNA targets.
